# Anus preservation in low rectal adenocarcinoma based on MMR/MSI status (APRAM): a study protocol for a randomised, controlled, open-label, multicentre phase III trial

**DOI:** 10.1186/s12885-024-11829-2

**Published:** 2024-01-10

**Authors:** Cheng-Yi Huang, Ming-Hua Bai, Jin-Wen Shen, Quan-Quan Sun, Yan-Ru Feng, Qian-Ping Chen, Wei Mao, Hai-Xing Ju, Ji Zhu

**Affiliations:** 1 Department of Radiation Oncology, Zhejiang Cancer Hospital, 310022 Hangzhou, Zhejiang China; 2 Postgraduate Training Base Alliance of Wenzhou Medical University (Zhejiang Cancer Hospital), 310022 Hangzhou, Zhejiang China; 3 Department of Colorectal Surgery, Zhejiang Cancer Hospital, 310022 Hangzhou, Zhejiang China; 4 Hangzhou Institute of Medicine (HIM), Chinese Academy of Sciences, 310022 Hangzhou, Zhejiang China; 5 Zhejiang Key Laboratory of Radiation Oncology, 310022 Hangzhou, China

**Keywords:** Rectal cancer, Anus preservation, Chemotherapy, Radiotherapy, Immunotherapy, MMR/MSI status, Neoadjuvant, W&W

## Abstract

**Background:**

Anus preservation has been a challenge in the treatment of patients with low rectal adenocarcinoma (within 5 cm from the anal verge) because it is difficult to spare the anus with its functioning sphincter complex under the safe margin of tumour resection. Patients with dMMR/MSI-H can achieve a favourable complete response (CR) rate by using a single immune checkpoint inhibitor. For patients with pMMR/MSS/MSI-L, intensified neoadjuvant three-drug chemotherapy may be the preferred option for anal preservation. In addition, the watch and wait (W&W) strategy has been proven safe and feasible for patients with rectal cancer who achieve a clinical complete response (cCR). Therefore, we initiated this clinical trial to explore the optimal neoadjuvant treatment pattern for patients with low locally advanced rectal cancer (LARC) with different MMR/MSI statuses, aiming to achieve a higher cCR rate with the W&W strategy and ultimately provide more patients with a chance of anus preservation.

**Methods:**

This is a randomised, controlled, open-label, multicentre phase III trial. Patients with clinical stage T2-4 and/or N + tumours located within 5 cm from the anal verge are considered eligible. Based on the results of pathological biopsy, the patients are divided into two groups: dMMR/MSI-H and pMMR/MSS. Patients in the dMMR/MSI-H group will be randomly allocated in a 1:1 ratio to either arm A (monoimmunotherapy) or arm B (short-course radiotherapy followed by monoimmunotherapy). Patients in the pMMR/MSS group will be initially treated with long-term pelvic radiation with concurrent capecitabine combined with irinotecan. Two weeks after the completion of chemoradiotherapy (CRT), the patients will be randomly allocated in a 1:1 ratio to arm C (XELIRI six cycle regime) or arm D (FOLFIRINOX nine cycle regime). The irinotecan dose will be adjusted according to the UGT1A1-genotype. After treatment, a comprehensive assessment will be performed to determine whether a cCR has been achieved. If achieved, the W&W strategy will be adopted; otherwise, total mesorectal excision (TME) will be performed. The primary endpoint is cCR with the maintenance of 12 months at least, determined using digital rectal examination, endoscopy, and rectal MRI or PET/CT as a supplementary method.

**Discussion:**

APRAM will explore the best anus preservation model for low LARC, combining the strategies of consolidation chemotherapy, immunotherapy, and short-course radiotherapy, and aims to preserve the anus of more patients using W&W. Our study provides an accurate individual treatment mode based on the MMR/MSI status for patients with low LARC, and more patients will receive the opportunity for anus preservation under our therapeutic strategy, which would transform into long-term benefits.

**Trial registration:**

Clinicaltrials.gov NCT05669092 (Registered 28th Nov 2022).

**Supplementary Information:**

The online version contains supplementary material available at 10.1186/s12885-024-11829-2.

## Introduction

Over the last 20 years, neoadjuvant CRT (NACRT) followed by total mesorectal excision (TME) has become the standard treatment for patients with locally advanced rectal cancer (LARC). This mode results in a significant reduction in local recurrence (LR) and improvement in anus function conservation patients with LARC, and a certain proportion of patients achieve a pathological complete response (pCR) [1, 2]. In the context of the elevation of clinical efficacy and demands for quality of life, the watch and wait (W&W) strategy has received extensive attention from the medical community. For patients with low rectal cancer who have difficulty achieving anus preservation by surgical resection, W&W can be adopted if they achieve a clinical complete response (cCR) after NACRT, and surgery becomes a salvage treatment.

In February 2014, senior clinical experts worldwide jointly constructed the International Watch and Wait Database (IWWD) to provide clinical evidence for W&W [3]. Data published in July 2018 revealed that the 2-year local regrowth rate, 3-year distant metastasis rate, and 5-year overall survival rate of 880 patients with cCR adopting W&W strategies were 25.2%, 8.1%, and 84.7%, respectively. Local tumour regrowth mainly occurs in the first 2 years after the completion of treatment, and 97% of local regrowth is located in the bowel wall [4]. The results of the IWWD updated in 2021 indicate that the risk of local recurrence in patients who maintain cCR for three years is less than 5%, and the risk of systemic recurrence is even lower [5]. This shows that with the extension of W&W time, the probability of local regrowth will gradually decrease, and the patient will eventually reach the “cure” state, which confirms the safety and feasibility of W&W.

The pCR rate in patients with LARC receiving traditional fluorouracil-based CRT is only 10–15% [2, 6]. The launch of this trial led to a national multicentre phase III CinClare clinical trial (NCT02605265) in China. This proved that conventional CRT based on capecitabine combined with different doses of irinotecan (CapIriRT regimen) under the guidance of the UGT1A1 genotype could double the pCR rate in patients with LARC (17% vs. 33%) [7, 8]. To further elevate the complete response (CR) of low LARC, this study focused on the following three aspects.

### Sequential consolidation chemotherapy after CRT

To achieve a better tumour response, the American OPRA study and German CAO/ARO/AIO-12 study compared the strategies of induction and consolidation chemotherapy [9, 10]. Both studies showed that consolidation chemotherapy had the advantages of better tumour response, higher organ preservation rates, less toxicity, and higher treatment completion than induction chemotherapy.

### Intensified three-drug chemotherapy

In view of the high objective response rate (ORR) and long progression-free survival (PFS) of combination chemotherapy regimens incorporating irinotecan in metastatic colorectal cancer, an increasing number of researchers have used three-drug chemotherapy as a neoadjuvant therapeutic strategy for LARC. In the PRODIGE23 study, patients with LARC treated with FOLFIRINOX induction chemotherapy showed higher pCR (28% vs. 12%) and 3-year disease-free survival (DFS) rates (76% vs. 69%) than those treated with traditional capecitabine-based NACRT [11]. Thus, more patients can have their anus preserved because of the higher pCR rate with three-drug chemotherapy.

### Combination with immunotherapy

In colorectal cancer, patients with dMMR/MSI-H can achieve an ideal pCR rate with a single immunotherapy. The NICHE study showed that a 60% pCR rate could be achieved through one cycle of neoadjuvant Nivo + Ipi treatment [12]. In the PICC study, patients with dMMR/MSI-H LARC achieved a decent pCR rate (88% vs. 65%) using the toripanimab +/- celecoxib regime [13]. Another study published in the New England Journal showed that 12 patients with dMMR/MSI-H LARC achieved cCR after dostarlimab monotherapy for 6 months [14]. However, in the KEYNOTE-177 study, nearly 30% of patients with dMMR/MSI-H advanced colorectal cancer did not respond to single immunotherapy [15]. Because of the small sample size and short follow-up time, the results need to be further validated.

The combination of radiotherapy and immunotherapy is promising. Radiotherapy can promote the immune system to recognise tumour antigens, activate the innate immune response, up-regulate the expression of PD-L1 in tumour cells, and transform the tumour microenvironment from immune-suppressed to immune activated. Immunotherapy promotes tumour vascular normalisation, improves hypoxia, enhances radiosensitivity, and regulates the tumour immune microenvironment [16]. Therefore, radiotherapy combined with immunotherapy may exert a synergistic effect.

In summary, under the current situation of clinical diagnosis and treatment of LARC and the trend of patient demand for anus preservation, we launched this randomised, controlled, open-label, multicentre phase III study based on the CinClare study [7]. This study aimed to explore the best neoadjuvant therapy mode for anal preservation in patients with LARC with different MMR/MSI statuses.

## Methods and analysis

### Study design

This randomised, controlled, open-label, multicentre phase III trial is scheduled to be conducted at Zhejiang Cancer Hospital, Hangzhou, China, between 1 January 2023 and 31 December 2025. This study is based on CinClare combined with different sequential modes of CRT, aiming to further explore the best strategy for cCR after neoadjuvant therapy for patients with LARC under different MMR/MSI statuses.

### Study objective

The primary objective of this study includes two aspects: (1) to explore whether patients can achieve a higher cCR rate using FOLFIRINOX three-drug consolidation chemotherapy compared to XELIRI two-drug consolidation chemotherapy following irinotecan-based NACRT for pMMR/MSS rectal cancer. (2) to explore whether patients can achieve a higher cCR rate using short-course radiotherapy followed by immunotherapy compared to immunotherapy alone for dMMR/MSI-H rectal cancer. The secondary objectives of this study are to evaluate the treatment-related toxicity, quality of life, and long-term prognosis in each group (local control (LC), overall survival (OS) and DFS).

#### Inclusion criteria

1. Rectal adenocarcinoma confirmed by pathology;
2. Baseline clinical stage T2-4 and/or N + on pelvic magnetic resonance images, not suitable to achieve radical resection by initial local resection;
3. Distance from anal verge ≤ 5 cm, or surgically assessed to be unable to directly perform sphincter preservation surgery;
4. Strong desire to preserve anus, and can receive at least 2 years of close monitoring after CRT;
5. No distant metastasis;
6. Age 18–70 years old, gender not limited;
7. ECOG PS 0–1;
8. Detection of UGT1A1*6 and *28 gene status (for pMMR patients);
9. Sufficient bone marrow reserve and physical ability to undergo consolidation chemotherapy after CRT (for pMMR patients);
10. Able to comply with the plan during the study period;
11. Signing a written informed consent.

#### Exclusion criteria

1. Pregnant or lactating women;
2. History of uncontrolled epilepsy, central nervous system diseases, or mental disorders judged by researchers who may hinder the signing of informed consent or affect patient compliance with oral drugs;
3. Difficult to achieve complete response at the level of available evidence, such as: maximum diameter of tumour > 10 cm; maximum diameter of lateral lymph nodes > 2 cm; baseline CEA > = 100 µg/L; signet ring cell carcinoma in biopsy pathology; or circumferential constriction tumour confirmed by digital rectal examination, which should be decided by the evaluation team if necessary;
4. Clinically severe (i.e., active) heart diseases, such as symptomatic coronary heart disease, New York Heart Association (NYHA) II or more severe congestive heart failure, severe arrhythmias requiring drug intervention, or a history of myocardial infarction within the last 12 months;
5. Organ transplantation needing immune suppressive therapy;
6. Severe uncontrolled recurrent infections or other serious uncontrolled concomitant diseases;
7. The baseline blood routine and biochemical indexes of the subjects not meeting the following criteria: haemoglobin level ≥ 90 g/L, absolute neutrophil count (ANC) ≥ 1.5 × 10^9^/L, platelet count ≥ 100 × 10^9^/L, ALT or AST level ≤ 2.5 times the upper limit of normal, ALP level ≤ 2.5 times the upper limit of normal, serum total bilirubin level < 1.5 times the upper limit of normal, serum creatinine level < 1 time the upper limit of normal, serum albumin level ≥ 30 g/L;
8. Known to suffer from dihydropyrimidine dehydrogenase (DPD) deficiency;
9. Allergic to any research drug ingredient.

#### Withdrawal

1. Use of other treatments simultaneously, which may impact the evaluation;
2. Use of the study drug inconsistent with the clinical protocol;
3. Not suitable to continue the treatment in case of serious adverse events;
4. Poor compliance of treatment;
5. In case of disease progression during treatment, other treatment strategies need to be adjusted in time after MDT (Multidisciplinary Team) discussion.

### Treatment

**Fig. 1:**
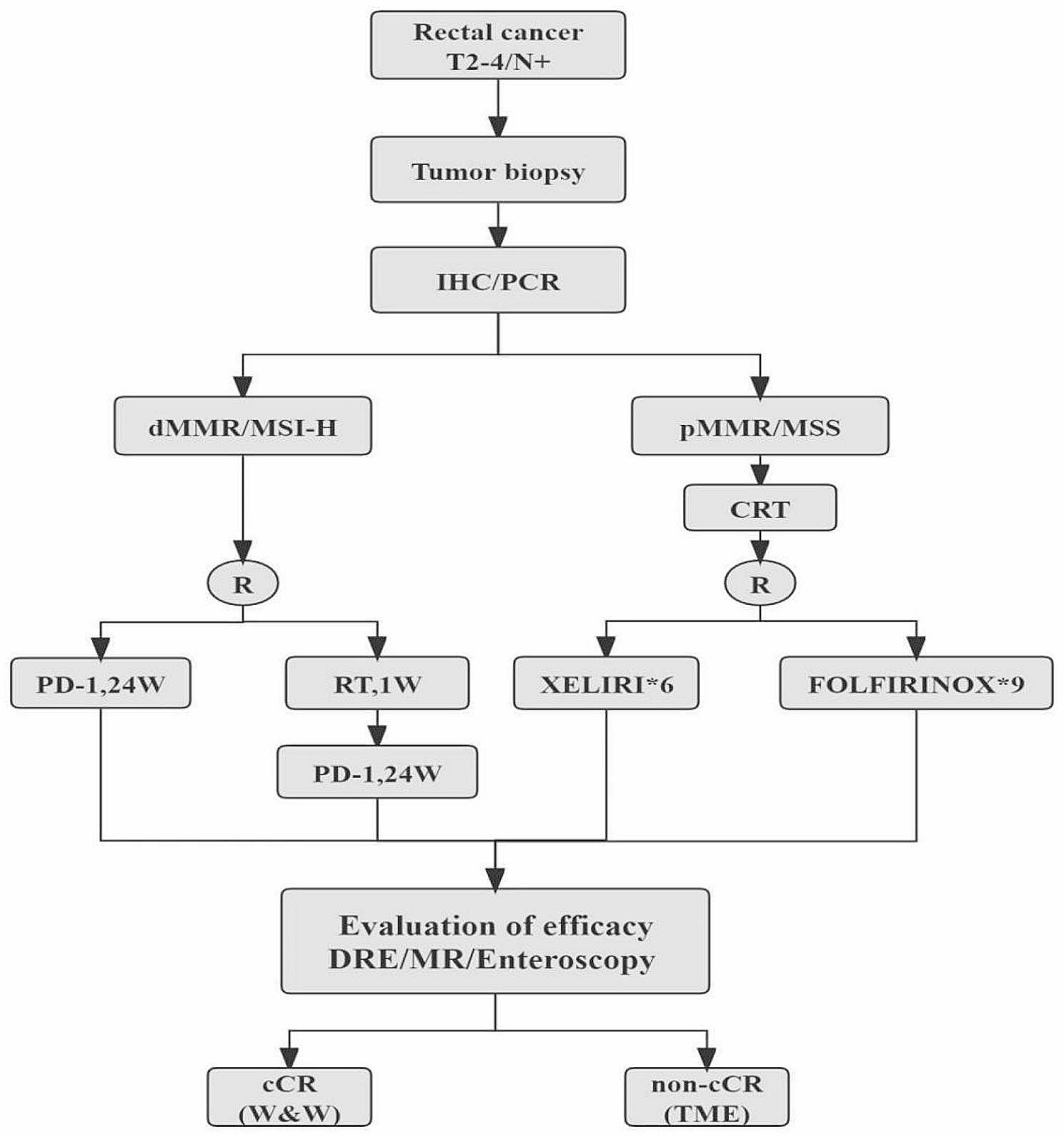
Flow chart of the study. IHC, immunohistochemistry; PCR, polymerase chain reaction; CRT, chemoradiotherapy; RT, radiotherapy; DRE, digital rectal examination; MR, magnetic resonance; TME, total mesorectal excision; W&W, watch and wait; XELIRI, capecitabine + irinotecan; FOLFIRINOX, 5-fluorouridine + irinotecan + oxaliplatin + leucovorin

This is a randomised, controlled, open-label, multicentre phase III trial. All eligible patients with rectal cancer undergo pathological biopsy to detect the MMR/MSI status (dMMR/MSI-H or pMMR/MSS) by IHC/PCR. Based on the detection results, patients are divided into two groups (dMMR/MSI-H and pMMR/MSS). The overall landscape is shown in Fig. 1.

In the dMMR/MSI-H group, patients are randomly assigned in a 1:1 ratio to arm A (monoimmunotherapy) or arm B (short-course radiotherapy followed by monoimmunotherapy).

In the pMMR/MSS group, the patients are first treated with long-course pelvic radiation with concurrent capecitabine combined with irinotecan. Two weeks after the completion of CRT, patients with adequate bone marrow reserves and the physical ability to receive subsequent therapy are randomly assigned in a 1:1 ratio to arm C (XELIRI group) and arm D (FOLFIRINOX group). Patients in arm C will receive treatment with XELIRI for six cycles, and those in arm D will receive treatment with FOLFIRINOX for nine cycles.

Within 2 weeks after all treatment completion, tumour complete response assessment will be performed in all patients using digital rectal examination, endoscopy, and rectal MRI. Patients who achieve initial cCR will undergo W&W, and those who do not achieve initial cCR will undergo TME.

### Research evaluation

#### Evaluation of toxicity and life quality

The toxicity is evaluated by National Cancer Institute Common Toxicity Criteria, version 4.0. The life quality is evaluated using Eq. 5D and EORTC QLQ -C30 (V3.0) scales.

#### Evaluation of efficacy

Patients are regularly examined during and after treatment to evaluate treatment efficacy. A baseline comprehensive assessment will be performed in all patients. A comprehensive assessment includes, digital rectal examination (DRE), tumour markers (CEA and CA199, etc.), chest CT, abdomen CT, pelvic MR and endoscopy. After radiotherapy completion, DRE, tumour markers, chest CT, abdomen CT and pelvic MR will be performed in the pMMR/MSS group. During systemic treatment, DRE will be performed once a month and tumour markers, chest CT, abdomen CT and pelvic MR examination will be performed once every two months. All patients will be scheduled to receive overall assessment within 2 weeks after all the treatments completion. For patients who do not achieve cCR, a comprehensive assessment will be performed before surgery. For patients who achieve cCR, W&W will be taken. A dedicated imaging assessment team composed of professional radiologists will review and confirm each image to ensure diagnostic consistency and accuracy.

#### Follow-up

For patients undergoing surgical resection, follow-up is in accordance with previous studies [7, 17]. For patients managed by watch and wait, an intensive follow-up protocol is used. In the first 12 months from the initial diagnosis of cCR, patients will be scheduled to receive DRE monthly, tumour markers, chest CT, abdomen CT, pelvic CT/MR every three months, and endoscopy examination yearly. In the second year, patients will be scheduled to receive DRE, tumour markers, chest and abdomen CT, pelvic CT/MR every three months and endoscopy examination yearly. In the next 3 years, follow-up investigations will be scheduled every six months and annually thereafter. The examination parameters and time points are listed in the Table 1.

**Table 1:** Treatment evaluation before, during and 2 years after the treatment completion

	Baseline before treatment	During radiotherapy (weekly)	During systemic treatment (every two months)	Patients who do not achieve cCR (before the operation)	Follow-up (every three months)
Physical examination	X	X	X(monthly)	X	X (patients with cCR receive DRE once a month in the first year)
Blood routine	X	X	X(weekly)	X	
Liver and kidney function	X	X	X(weekly)	X	
Tumour marker	X		X	X	X
Chest CT	X		X	X	X
Abdomen CT	X		X	X	X
Pelvic MR	X		X	X	CT/MR
Endoscopy ± endoscopic ultrasonography	X			If necessary	Once a year
Quality of life evaluation	X		X	X	X
Toxicity evaluation	X	X	X	X	X

DRE: digital rectal examination

#### Definition of cCR

We use internationally recognised criteria [18, 19] to define initial clinical complete response (cCR).

- Endoscopic: white scar, teleangiectasia, absence of ulceration and/or mass.
- DRE: no irregularity, firm area with minor induration.
- Radiological: mrTRG1: fibrosis with low signal intensity seen on T2 weighted images replacing the primary tumour; no restricted diffusion on diffusion weighted images; no nodes with border irregularity or mixed signal intensity; no extramural vascular invasion.

If the DRE, pelvic MR and endoscopy (combined with PET-CT if necessary) can jointly ascertain no visible tumour residue, and the status of tumour free can maintain for more than 12 months, it can be considered to achieve cCR.

#### Definition of near cCR

A clinical near-complete response (near cCR) is defined as follows:

- Endoscopic: a small residual flat ulcer, or irregular wall thickening at endoscopy and/or dysplasia at histopathology. Biopsy is mandatory.
- DRE: a superficial soft irregularity.
- Radiological: an obvious downstaging with/without residual fibrosis, but with a heterogeneous or irregular aspect on T2 weighted images and/or a small focal area of restricted diffusion on diffusion weighted images.

For patients achieving near cCR after overall assessment, they will undergo TME or local excision according to the evaluation of experienced surgeons.

### Drug regimens

In arm A (monoimmunotherapy), patients will receive 3 mg/kg triprilizumab iv on day 1 per 14 days for a total of 24 weeks. In arm B (short-course radiotherapy followed by monoimmunotherapy), patients will receive short-course radiation therapy (pelvic radiation of 25 Gy/5 in fractions) firstly. One week after radiation, patients will receive 3 mg/kg triprilizumab iv on day 1 per 14 days for a total of 24 weeks.

In the pMMR/MSS group, eligible patients will first be treated with concurrent CRT, with the specific program as follows: pelvic radiation of 50 Gy/25 fractions delivered with a 6–10 MV photon beam via intensity-modulated radiation therapy; capecitabine: 625 mg/m^2^ bid on day 1–5 per week; irinotecan: the dosage will be determined according to the UGT1A1*6 and *28 genotypes. (1) Total wild type (UGT1A1 genotypes G/G and 6/6): 80 mg/m^2^/week for 5 weeks. (2) Single-locus mutation (UGT1A1 genotype G/G and 6/7 or G/A and 6/6): 65 mg/m^2^/week for 5 weeks. (3) Double locus mutation (UGT1A1 genotype of G/A and 6/7 or A/A and 6/6 or G/G and 7/7): 50 mg/m^2^/week, at weeks 1, 2, 4, and 5 for a total of 4 weeks. After concurrent CRT, patients in arm C will receive 1000 mg/m^2^ capecitabine bid on day 1–14 combined with 200 mg/m^2^ irinotecan ivgtt on day 1 per 3 weeks for a total of six cycles. In arm D, patients will receive 150 mg/m^2^ irinotecan ivgtt on day 1 (double locus mutation: reduction to 120 mg/m^2^), 85 mg/m^2^ oxaliplatin ivgtt on day 1, and 2400 mg/m^2^ 5-FU ivgtt 46 h per two weeks for a total of nine cycles.

When the following occurs, combined use of drugs is needed:

1. G-CSF can be used when neutropenia occurs in degree 2–4;
2. When fever is caused by neutropenia, antibiotics should be administered when G-CSF is used;
3. TPO or IL-11 can be used when thrombocytopenia occurs in degree 2–4;
4. Component transfusions can be performed if necessary; however, erythropoietin (EPO) is not recommended.

### Study end point

The primary endpoint is a clinical complete response (cCR). The definition of cCR is presented in details above. Secondary endpoints are treatment-related toxicity (National Cancer Institute Common Toxicity Criteria, version 4.0), quality of life (EORTC-C30, QC5D), organ preservation rate, LC, DFS, and OS. Survival time is calculated from the date of randomisation to the date of the event or last follow-up. Events are defined as local failure of local control, tumour recurrence or death from any cause for DFS, and death from any cause for OS.

### Sample size

For the pMMR/MSS population, the organ preservation rate is expected to increase from 40% [7] in the XELIRI consolidation group to 70% in the FOLFIRINOX consolidation group. Each group requires 60 patients, for a total of 120 patients. A total of 132 patients will be recruited, considering a 10% loss to follow-up.

In the dMMR/MSI-H population, it is expected that the organ preservation rate will increase from 55% [13, 15] in those administered simple PD-1 inhibitors to 90% in the combined radiotherapy group (α = 0.05, power = 0.80). Each group will require 19 patients, for a total of 38 patients. A total of 42 patients will be recruited, considering a 10% loss to follow-up.

### UGT1A1 genotype examination

The details are presented in the supplementary materials.

### Statistical analysis

If the continuous variable accords with the normal distribution, it will be expressed by the mean +/-standard deviation, and if it does not accord with the normal distribution, it will be expressed by the median. The classified variable is described by frequency. The means of the two groups will be compared using the t-test, and the rates will be compared using the chi-squared test. The local control and survival rates for long-term prognosis will be evaluated using the Kaplan-Meier method and compared using the log-rank method. A statistically significant difference will be defined as *P* < 0.05.

### Safety

Any adverse medical event from the time the patient signed the informed consent form and enrolled in the study until the last visit, regardless of whether there was a causal relationship with the drug studied, will be determined to be an adverse event. Adverse events are regarded as serious adverse events (SAE) when they meet one or more of the following criteria: death, life-threatening adverse events, requiring or prolonging hospitalisation, persistent or severe disability or insufficiency, congenital malformations or birth defects, and major medical events. Once SAE occurs in patients, all anti-tumour therapies should be stopped immediately, and the relationship between adverse events, anti-tumour drugs, and radiotherapy should be evaluated. The corresponding symptomatic support treatment should be administered until the patient is cured or the condition is stable.

## Discussion

The W&W strategy has been confirmed to be safe and feasible for patients with low LARC who have achieved cCR after neoadjuvant CRT and has great significance for anus preservation [3–5]. In the CinClare study, the pCR rates reached 33% in the experimental group treated with irinotecan-based CRT under the guidance of the UGT1A1 genotype, compared to 20% in another phase III study, ARISTOTLE, during the same period. However, the pCR rate of 33% is still low for LARC, especially in patients who hope to preserve the anus with neoadjuvant CRT [7]. There is strong evidence indicating that patients with dMMR/MSI-H can achieve a desirable CR rate with monoimmunotherapy [12, 13]. In this study, short-course radiation followed by PD-1 monotherapy in the dMMR/MSI-H group are conducted to achieve a synergistic effect and attain a higher cCR rate than monotherapy with PD-1. Concurrent NCRT is the standard treatment for patients with a pMMR/MSS. Concurrent CRT followed by consolidation chemotherapy has been validated to achieve better tumour response than induction chemotherapy in the OPRA [9] and CAO/ARO/AIO-12 studies [10]. In this study, we aim to identify the best consolidation regimen between XELIRI and FOLFIRINOX and explore the best chemotherapy mode for anus preservation in patients with low LARC.

In conclusion, to further elevate the cCR rate for low LARC, based on a previous study and comprehensive consideration, we design this study combining the strategies of consolidation chemotherapy, immunotherapy, and short-course radiotherapy, aiming to preserve the anus of more patients using W&W. Our study provides an accurate individual treatment mode based on the MMR/MSI status for patients with low LARC, and more patients will receive the opportunity for anus preservation under our therapeutic strategy, which would transform into long-term benefits.

### Electronic supplementary material

Below is the link to the electronic supplementary material.

Supplementary Material 1

## Data Availability

Not applicable.

## Abbreviations

CR: Complete response
cCR: Clinical complete response
pCR: Pathological complete response
LARC: Locally advanced rectal cancer
W&W: Watch and wait
dMMR/MSI-H: Deficient mismatch repair/Microsatellite instability-High
pMMR/MSS/MSI-L: Proficient mismatch repair/ Microsatellite stability/ Microsatellite instability-Low
OS: Overall survival
LR: Local recurrence
LC: Local control
DFS: Disease-free survival
ORR: Objective response rate
PFS: Progression-free survival
MRI: Magnetic resonance imaging
PET-CT: Positron emission tomography/computed tomography
PD-1: Programmed cell death-1
Nivo: Nivolumab
Ipi: Ipilimumab
CRT: Chemoradiotherapy
NACRT: Neoadjuvant chemoradiotherapy
DRE: Digital rectal examination
TME: Total mesorectal excision
MDT: Multidisciplinary team
DPD: Dihydropyrimidine dehydrogenase
IHC: Immunohistochemistry
PCR: Polymerase chain reaction
SAE: Serious adverse events
