# Supplementary material for: Anus preservation in low rectal adenocarcinoma based on MMR/MSI status (APRAM): a study protocol for a randomised, controlled, open-label, multicentre phase III trial
Source: BMC Cancer. 2024 Jan 10;24:57. doi: 10.1186/s12885-024-11829-2 (PMC10782729; doi:10.1186/s12885-024-11829-2)
Supplement: Supplementary file 1 — Supplementary Material 1 [file 12885_2024_11829_MOESM1_ESM.docx]

**UGT1A1 Genotyping**

All patients will undergo UGT1A1 genotyping. Before treatment, 2 mL of venous blood will be collected, and blood samples will be collected using DNA extraction kits (QIAamp DNA Blood Midi Kit; QIAGEN, Venlo, the Netherlands). Polymerase chain reaction assay is performed in a 25-uL reaction with 2.5 uL of 15 mM Mg^2+^, 2 uL of 2.5 mM deoxyribonucleotide triphosphates, 5 U Taq, and 30 ng of DNA. The following primers are used: UGT1A1*6-forward, GACGCCTCGTTGTACATCAGA and UGT1A1*6-reverse, CACGCTGCAGGAAAGAATC; UGT1A1*28-forward, TGAACT -CCCTGCTACCTTTGTG and UGT1A1*28-reverse, TCCACTGGGATCAACAGTAT -CTT. The reaction is run for 35 cycles at 95°C for 30 seconds, 60°C for 30 seconds, and 72°C for 30 seconds. Genotypes are assigned on the basis of the number of thymine-adenine repeats in each allele.
